# Designing and interpreting ‘multi-omic’ experiments that may change our understanding of biology

**DOI:** 10.1016/j.coisb.2017.08.009

**Published:** 2017-12

**Authors:** Robert Haas, Aleksej Zelezniak, Jacopo Iacovacci, Stephan Kamrad, StJohn Townsend, Markus Ralser

**Affiliations:** 1 The Molecular Biology of Metabolism Laboratory, The Francis Crick Institute, London, NW1 1AT, United Kingdom; 2 Department of Biochemistry and Cambridge Systems Biology Centre, University of Cambridge, Cambridge, CB2 1GA, United Kingdom; 3 Department of Biology and Biological Engineering, Chalmers University of Technology, SE-412 96, Göteborg, Sweden; 4 Department of Surgery and Cancer, Division of Computational and Systems Medicine, Imperial College London, London, SW7 2AZ, United Kingdom; 5 Department of Genetics, Evolution and Environment, University College London, WC1E 6BT, London, United Kingdom

## Abstract

Most biological mechanisms involve more than one type of biomolecule, and hence operate not solely at the level of either genome, transcriptome, proteome, metabolome or ionome. Datasets resulting from single-omic analysis are rapidly increasing in throughput and quality, rendering multi-omic studies feasible. These should offer a comprehensive, structured and interactive overview of a biological mechanism. However, combining single-omic datasets in a meaningful manner has so far proved challenging, and the discovery of new biological information lags behind expectation. One reason is that experiments conducted in different laboratories can typically not to be combined without restriction. Second, the interpretation of multi-omic datasets represents a significant challenge by nature, as the biological datasets are heterogeneous not only for technical, but also for biological, chemical, and physical reasons. Here, multi-layer network theory and methods of artificial intelligence might contribute to solve these problems. For the efficient application of machine learning however, biological datasets need to become more systematic, more precise – and much larger. We conclude our review with basic guidelines for the successful set-up of a multi-omic experiment.

## Highlights

• Most biological mechanisms involve more than one class of biomolecule.
• Multi-omics approaches integrate information from multiple layers of biological data.
• The bottleneck remains in low sample numbers and strategies to make sense out of heterogeneous datasets.
• Multi-layer networks and artificial intelligence can dissect multi-omics data if large and systematic datasets are available.

## Introduction

**Figure 1:**
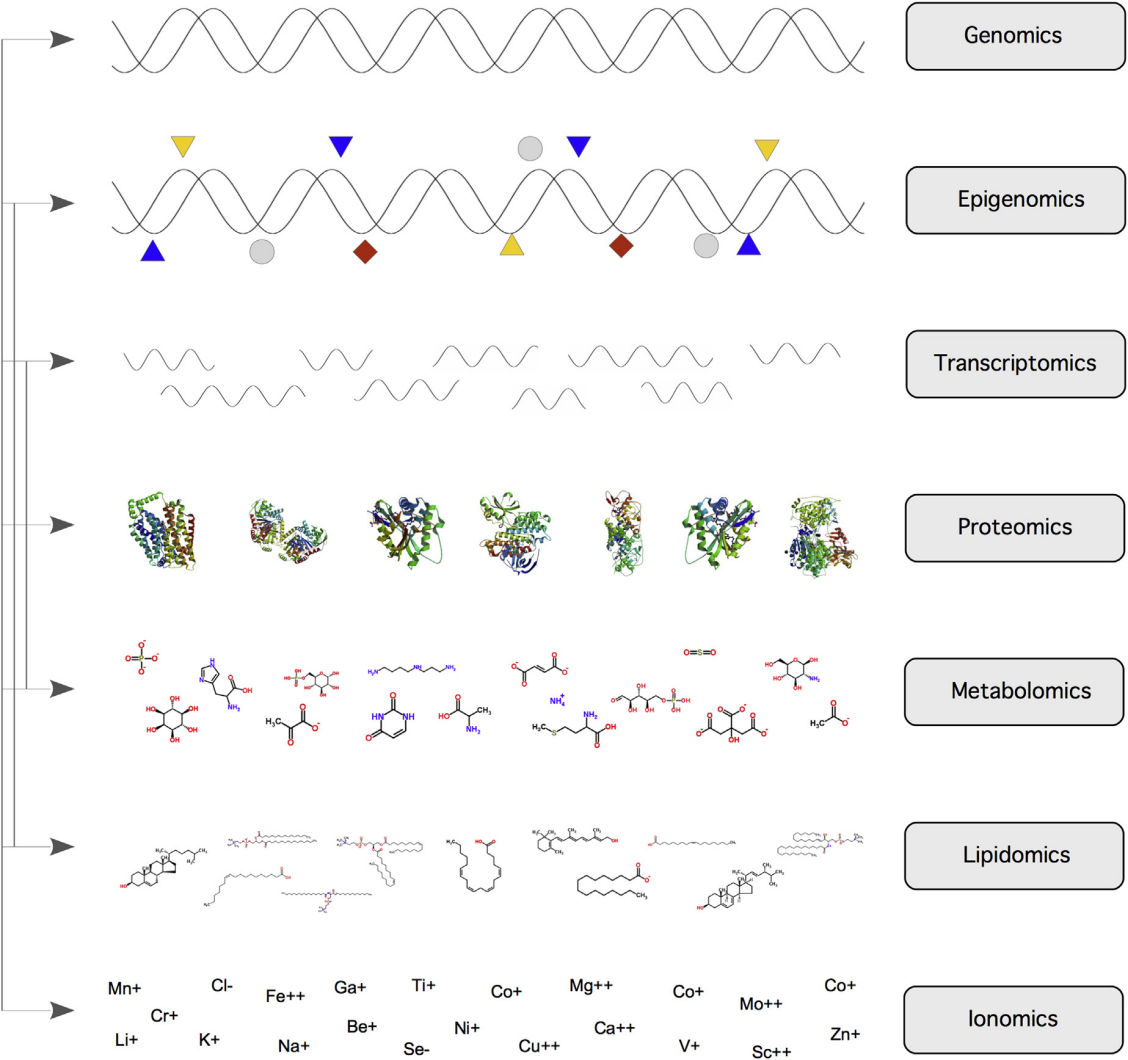
Overview of cellular layers and omic technologies to analyse them. The different layers of the cell, consisting of DNA sequence and modifications (Genome, Epigenome), RNA and protein content (Transcriptome, Proteome) small molecules (Metabolome, Lipidome) and elemental composition (measured as ‘Ionome’), can be analysed using according omic technologies (right). The combination of omic layers in a multi-ome dataset is able to reveal inter-layer mechanisms that would otherwise be left concealed by each single layer in separation. The figure illustrates the hugely divergent chemical make-up and complexity of each layer. Arrows illustrate the degree of dependency between the levels.

Many biological processes are highly dynamic, and their regulation as well as functionality involves a multitude of interactions between the genome, epigenome, transcriptome, proteome, metabolome, and ionome 1, 2, 3. Thus, in order to comprehensively understand a process of fundamental biological importance, it is critical not only to understand these biological layers as separate elements, but to dissect how they interact with one another (Figure 1).

An unprecedented pace in the development of ‘omic’ technologies, as well as increasing investments into research facilities, has greatly increased access to ‘genome-scale’ technologies across the biosciences. Studies involving multiple of these techniques (‘multi-omics’) have given rise to a new era in Systems Biology, but generate the need to integrate and combine very different types of biological information. While the obvious need for a ‘multi-level’ biological analysis has created an anticipation that multi-omics is capable of revealing new biological mechanisms, it is becoming increasingly clear that our current methodological spectrum for the analysis of biological data, and the theoretical framework required to interpret the obtained information, is lagging far behind. Hence, a large number of high-quality datasets are created, only to be incompletely analysed, and lots of biological insight remains buried. In this review we highlight some new developments that aim to change this situation, and discuss typical pitfalls that are to be avoided in order to conduct a successful multi-omic study.

## Challenges to combine multi-omic biological information

While none of the current omic technologies is perfect, some come considerably closer to providing a comprehensive picture of the biological layer they aim to address, whilst some others lag behind. Often, this has less to do with the state of the technological developments themselves, and more with huge differences in the chemical and physical complexity of each biological level (Figure 1).

1. **The genome**, according to the *Central Dogma* [4], is the basal layer of the cell, and, at the same time, it is the biological layer most effectively captured by current omic technology. Being composed of strands of the four nucleotides, the genome is a linear, effectively digital sequence. By leveraging the intrinsic complementarity of base pairs (sequencing by synthesis), it has become possible to rapidly genotype an unprecedented number of samples at a relatively low cost 5, 6, 7, 8. The ability to efficiently sequence genomes and to predict RNA and protein sequences from it 5, 6, 7, 8, has effectively opened the door for multi-omic approaches. The digital nature of the DNA sequences renders them the easiest form of biological ‘omic’ information to be stored in databases and shared between labs.
DNA sequence information is static by nature and is not directly informative about biological mechanisms encoded within it. ‘Epigenomics’, the genome-wide picturing of DNA modifications or chromatin structure in 2D and 3D 9, 10, 11, is progressing rapidly, but is not yet covering the comprehensive set of DNA modifications or structural elements. In order to get a comprehensive view on cellular heterogeneity, biological problems are more often investigated on a single cell level. For an excellent review about the state of te technology and remaining challenges, the reader is referred to [12].
2. **The transcriptome** was the first ‘functional’ molecular layer of the cell that was accessible on the genomic scale, and remains the dynamic layer with the best coverage [13]. Indeed, the rise of transcriptomics led to a plethora of biological discoveries, and transcriptomic was the technology that opened the door for the first series of real multi-omic studies, where comparisons between DNA sequence and mRNA expression facilitated the identification of structural elements in genome and transcriptome [14]. Transcriptional analysis remains therefore more frequently employed – i.e. it is for most biologists the first contact with an ‘omic’ technology – as its data is still more easily analysed and shared than the more ‘downstream omics’ such as proteomics and metabolomics. More recently, transcriptomics is enjoying a second revival, as it is in many cases applicable to single cells 15, 16, 17, 18.
3. **The proteome** is the primary ‘functional’ layer of the cell bridging gene expression to phenotype, and therefore of massive complexity 19, 20. While the sequence of a protein can be (largely) derived from genome and transcriptome, the function of a protein depends on its concentration, folding, turnover, post-translational modifications, cellular localisation, and its binding to other proteins and metabolites. As a consequence of this complexity, no technology covers the proteome in its diversity comprehensively. While most proteins can now routinely be quantified in a low number of samples, post-translational modifications and dynamic structural changes still fall short of being exhaustively quantifiable 21, 22, 23, 24, 25, 26. Further challenges for the era of data driven biology consider sample throughput, that in proteomics remains considerable lower as in genomics, transcriptomics or metabolomics, and quantitative precision on large sample series, that is – compared to the other omic-technologies – low.
4. **The metabolome** is the first cellular layer that is not directly encoded in the genome, but is instead a product of the functional spectrum of the proteome, in contact with the environment of the cell [27]. Therefore, the metabolome constitutes a ‘phenotype’ of the cell. Despite being downstream, the genome, transcriptome and proteome consist however of components made by the metabolome. Furthermore, the central components of the metabolome are better conserved across all organisms compared to genome, transcriptome and proteome, and can also be recapitulated by a non-enzymatic chemistry, and hence the metabolome is believed evolutionarily to be the oldest part of the cell 28, 29. The metabolome was recognized as a key player in very early clinical research, a similar central role in the molecular biosciences has recently begun to be re-instated 30, 31. Due to its enormous chemical complexity, the metabolome can however not be captured by a single technology comprehensively. However, efforts in reconstructing metabolic networks on the basis of the rules of biochemistry, have been fruitful on the cellular scale. Genome-scale reconstructions of the metabolic network form the basis for the prediction of cellular phenotypes such as gene essentiality and the growth rate 32, 33, 34. Interactions between the metabolome and the proteome are considered key in the identification of so far overlooked cellular mechanisms. A key challenge to generate large-scale metabolomics data for the era of data driven biology, is to make the right decision between the need to quantify a low number of metabolites at high precision [35], or a large number of metabolites at lower precision [36], complementary approaches picturing a different set of biological mechanisms.
5. **The ionome** reflects total elemental composition of the cell 37, 38. Different to other omic layers, the ionome is not produced by the cell, but rather is a consequence of transport and diffusion processes and the incorporation of elements into biomass [39]. Therefore, the total and relative cellular ionome is sensitive to the genetics of an organism, but also all physiological changes with a significant impact on any form of membrane transport, intra- and extra-cellular pH, redox potential, ionic strength, nutrient supply, metabolic activity, cell size, membrane composition and potential, and changes in organellar biology. If significantly altered, the ionome is expected to have massive impact on the function of any biological system as it determines the cellular reaction environment, and with it, all simultaneously co-occurring chemical processes. As a result, the ionome represents the convergence of the physiological changes originating over genome, transcriptome, proteome and metabolome [40]. However, although the ionome can be precisely measured, the biological interest in ionomic data has remained moderate because the interpretation is so far challenging.
6. **The phenome** is the sum of all organismal phenotypes, representing the top layer of omic applications [41]. While each database or functional-genetic screen collecting phenotypic information is in essence a form of ‘phenomics’, several studies have attempted to achieve the systematic generation of ‘phenomes’. These include detection of growth size of bacterial or fungal colonies [42], the movement of *C. elegans* in different environments [43], or the non-invasive studies of growth and photosynthesis in plants [44], to name a few. The main challenge of phenomics lies in the recording of the vast possibilities of traits that emerge from the combination of influences from environmental and genomic cues, superimposed by cell-to-cell and temporal heterogeneity [41].

## The key challenge: how to render descriptive data predictable about function

It is noteworthy that many of the most successful applications of multi-omic studies did address the functionality of metabolism (some key papers are found here. ∗45, 46, ∗∗47, 48, ∗∗49, 50, 51) or very basic cellular processes such as translation 52, 53, 54 or transcription factor binding 55, 56 via integration of separate omics experiments.

This is perhaps less surprising in the sense that metabolic networks function in a cross-layer manner, and hence, depend on multi-omics data by principle. However, it is also of note that the field of metabolomic research has provided many research tools to facilitate the integration of multi-omic technologies. Moreover, the huge effort invested to reconstruct the topological organisation of the metabolic network on the genomic scale is starting to pay back 57, 58, 59. Nonetheless, despite these successes, neither metabolome, in addition to most downstream phenotypes, remains so far not predictable from multi-omic data ∗45, 50.

Why is the combination of different layers of biological data so complicated? Although none of proteome, metabolome, ionome and phenome can be fully captured at this time, the partial coverage that is already possible has resulted in staggering amounts of multidimensionality that is difficult to capture with the classically applied methods of biological data analysis [60]. New avenues are however enabled through network science, which has historically contributed to the study of omics data by shedding light on the topology and organisation of biological networks such as metabolic-reaction networks, protein-protein interaction networks and genetic regulatory networks 61, 62. Recently, a mathematical framework has emerged in network science which appears promising for the task of integrating multi-omic data: multi-layer networks 63, 64, 65. Multi-layer networks are capable of describing systems where interactions of different nature are involved. In its most general formulation, a multi-layer network is a network formed by several layers or standard networks. Each of these layers describes interactions/relations of a specific kind between the nodes of that layer.

**Figure 2:**
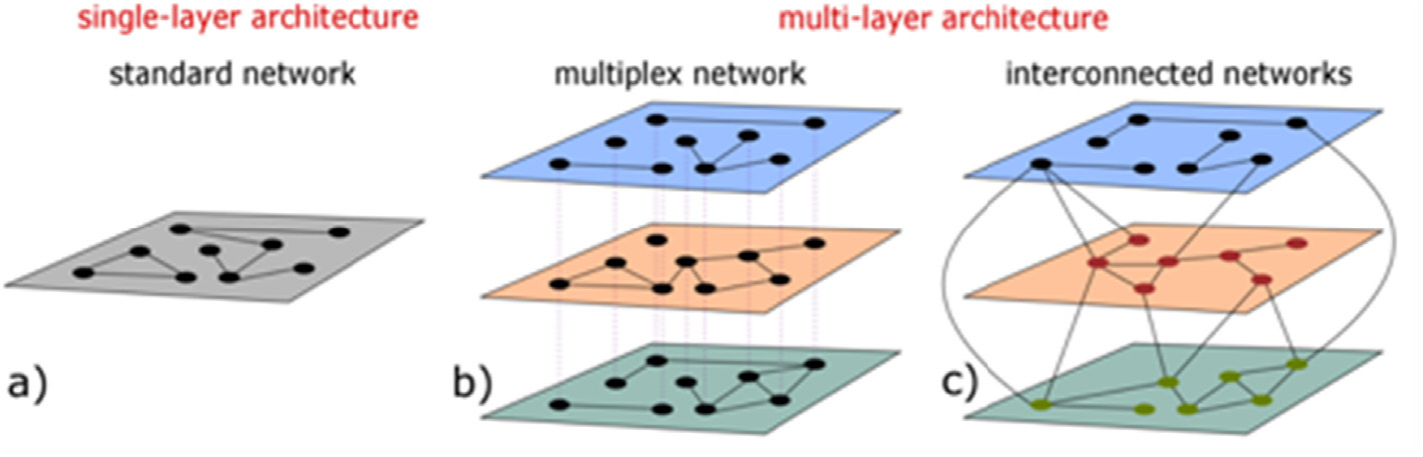
Different types of network architectures used in omics data analysis. a) a standard single-layer network can be used to describe single-omics datasets. b) a **multiplex network** is a multi-layer network formed by a unique set of nodes connected in several layers, each of them describing different types of interactions/relations. Multiplex networks can be used to study multi-omic data involving a specific class of biomolecules or different classes of biomolecules for which a one-to-one relation can be established (for example genes and proteins). c) an **interconnected network** is formed by several layers, each of them describing interactions/relations between a different set of nodes. Nodes joining different layers can be connected through inter-layer connections.

The first class of multi-layer networks are the multiplex networks [63]. A multiplex network comprises a common set of nodes but the pattern of connections between them is layer-specific (Figure 2B). Because the set of nodes is shared between the layers, these network structures are suitable for the analysis of multi-omics data featuring either a single class of biomolecules or different classes of biomolecules for which a one-to-one correspondence between the classes can be established. For instance, the multiplex network framework was successfully applied to analyse multi-omic datasets from different types of tissues (gastric, lung, pancreas, colorectal) under normal and cancer conditions by considering co-expression data, protein-protein physical interactions, transcription factor co-targeting relations, and microRNA co-targeting relations. A consensus clustering algorithm was used to reveal multi-layer communities and identify the candidate driver genes for the different types of cancers through an enrichment analysis of the communities [66].

In the second class – interconnected networks – nodes in different layers represent distinct types of objects, which can vary in number, and interactions between nodes from different layers can be described by inter-layer connections (Figure 2C). Interconnected networks can be used to model multi-omic data that involve biomolecules of different classes (e.g. proteomics and metabolomics data), and interactions/relations between biomolecules that belong to the same class (e.g. physical interactions between proteins or metabolic reactions between metabolites) or to different classes (e.g. proteins catalysing reactions between metabolites). For example, trans-omic networks are global biochemical networks that result from integrating measurements across the multiple omic layers of the genome, transcriptome, proteome and metabolome [65].

## Multi-layer networks as basis for the biological application of artificial intelligence

A multi-layer network representation containing all the functional interactions within and across all the omic layers of the cell for a given organism would fascinatingly and comprehensively describe our knowledge about that system. However, at the moment, we lack such a description because of the incompleteness of the data collected in the different omic fields and because of the limitations in the accuracy of the recorded and available data itself. This makes it necessary to incorporate in the multi-layer network models of the cell other mechanistic models and/or data-driven machine learning algorithms that are able to fill the gap in our empirical knowledge of how the information flows across the omic layers 67, 68, 69.

Artificial intelligence (AI) based machine learning approaches allow learning of complex functional relationships from data in an unbiased fashion without the need of *a priori* assumptions. The principle of a machine learning model is to train on one biological dataset and then use detected patterns to predict another. AI has been a great success in computer science and, in regards to biological approaches, is especially appealing for building predictive models on the basis of biological networks when underlying molecular mechanisms are unknown. Already, the multi-omics field has benefited greatly from machine learning, including for the analysis of genomics, proteomics and metabolomics data sets 70, 71, 72. Recent applications range from clinical predictions in cancer therapy to personalised dietary interventions based on prediction of postprandial glucose responses from DNA sequences obtained from stool microbiota [73].

Machine learning for typical biological applications can be divided into two major categories, namely, supervised learning and unsupervised learning. In supervised learning, the goal of machine learning algorithms is to learn a function *y* from the set of features *x*_n_ present in the training dataset. These features can represent any molecular signals, such as DNA sequence, expression of genes, proteins, metabolites or a set of pixels in imaging data. The response function y can be anything of interest spanning from the disease class to the levels of transcripts to be predicted from DNA features. Conversely, in unsupervised learning, the aim is to infer a function that describes a hidden structure from an “unlabeled” example, e.g. identify common molecular patterns that form a cluster of samples, typically used in “guilt by association” analyses for function prediction 35, 74.

The challenges associated with integrative analysis of multi-omics datasets arise from the inherent heterogeneity of the data. Any unsupervised learning technique is ultimately based on the study of variation between the samples. However, different types of datasets often have different numbers of features. In addition, the typical degree of feature variation depends strongly on the nature of the data, whilst conventional unsupervised methods, such as PCA for dimensionality reduction or K-means for clustering, are insensitive to features with low inter-sample variation. These methods thus cannot be directly employed for comprehensive analysis of concatenated datasets, in which multiple sets of features of different types are matched to the same samples. To address this problem, alternative statistical approaches are being developed to deal specifically with multi-omics data [75]. For example, the contribution of each feature set can be weighted using multiple factor analysis [76], or the features from different sets can be modelled using a common set of Gaussian latent variables [77].

Conventional supervised learning algorithms e.g. linear regression, logistic regression, support vector machine (SVM) and decision trees, require a set of manually engineered features that represent one input layer and allow for prediction of an output layer. Such architectures are typically called “shallow” and have been shown to be limited in their applications even when large datasets are available [78]. In contrast, “deep” architectures or “deep learning” [79] is abstracted by multiple hidden layers between input and output layer. In each layer the information is passed on to each unit as a weighted sum of units from previous layers with some – usually nonlinear – transformation in order to obtain a new representation of the input [80]. Such architectures benefit from very large datasets for finding structures hidden within them for learning complex feature representations that are successfully used to make accurate predictions out of biological data. Apart from accurate predictions, these “self-learned” feature representations have the potential of uncovering complex molecular interactions that would have been missed in a conventional hypothesis-driven paradigm. A recent review provides more specific examples about the applications of deep learning in biology [81].

One of the key remaining challenges is the interpretation of machine learning models. For instance, in biology, it is naive to be aiming for identification of a “key regulator” protein when, in reality, all processes are multifactorial. Similarly, in machine learning, predictive features are multidimensional and are usually in complex relationships with each other. As a result, one needs to be more careful when interpreting resultant models. To facilitate the interpretation of the machine learning models achieving a prediction – hence to use them to identify biological mechanisms – one can apply them to distinct biological networks. For instance, using metabolic networks one can integrate data to link gene expression to metabolism 50, 82, 83, or, one can apply them to phosphoproteomics data to predict metabolite concentrations from kinase activity profiles [84]. In a similar fashion one can abstract data to the known processes that are predictive of the outcome of interest. Applications of deep learning techniques are currently mostly limited to sequence and image analysis and will open its full potential when omics-technologies increase throughput significantly. For instance, with the development of single-cell technologies for other – omics, a single experiment will interrogate tens of thousands of individual cells opening new horizons in quantitative biology. A key problem here is the quantitative precision: artificial intelligence will only successfully detect the biological patterns from features that have a positive signal to noise ratio. While genomics, metabolomics and ionomics achieve high precision values on large datasets, single-cell mRNA sequencing and quantitative proteomics in particular need to improve in this respect. Once these problems are solved, the use of artificial intelligence in data driven systems biology through the integration of multi-omics will be a key next step in solving the genotype-phenotype problem.

## Conclusions

The remarkable advances in imaging, sequencing, and mass spectrometry technologies have made large scale omics datasets increasingly available. Despite the huge amount of accessible data generated by single-omic experiments, identification of novel biological mechanisms upon combining them has not yet reached expectations. This is partially caused by the intrinsic difficulties to combine highly heterogeneous data, and the fact that the supposedly ‘large’ datasets are still often small compared to what would be needed to work effectively using unsupervised learning approaches, and often fall short of the required precision. In order to achieve a high information content in multi-omic data, as a community we need to learn to adhere to standards, to conduct experiments in a highly systematic fashion, and to anticipate the integration of sophisticated mathematical approaches at the stage of experimental planning (Box 1). The analysis of multi-omic data via multi-layer networks and machine learning is highly promising. Biology is indeed in the process of obtaining a closer attachment to the data science. Multi-omic sciences has the potential to transform our understanding of biological systems and enable an excitingly fresh view on how the biological system is functioning.

Box 1 **The beginner's guide on the design of a multi-omic experiments**

The rapid development of omics technologies has meant that it is now financially and technically feasible for many research groups to perform not only single omics experiments, but to produce multiple omics datasets. We summarize here some key elements to consider when attempted to conduct a multi-omic experiment for the first time.

1. **Beware! The scientific part of multi-omic work starts, not ends, when the biological data is recorded.** Whilst seemingly obvious, we feel that this is an important point to make given the number of multi-omics datasets published without comprehensive follow-up data analyses. Furthermore, please be aware that it has somehow become typical in single omic analyses to set arbitrary thresholds to classify what is up or downregulated and to perform enrichment analysis, whilst sample comparison is often based on correlation coefficients, similarity heatmaps, principal component analysis or even Venn diagrams of up or downregulated genes. There is nothing wrong with this analysis *per se*, but similar analyses will not interpret multi-omic datasets due to their heterogeneous natures. So book onto an R-course, at least.
2. **Be prepared to spend a long, long time with the data to understand it.** On a related point, the power of multi-omics to generate mechanistic models comes from integration of the different omics layers. The value of the network, which can be constructed from multi-omics datasets is greater than that of the sum of the single omics layers analysed separately, as these networks often recapitulate the topology of the real biological network and hence can be used to construct mechanistic and predictive models of biological phenomena. This means, however, one does enter uncharted territory and one typically cannot ‘outsource’ the interpretation of a multi-omic dataset to someone else, i.e. a bioinformatics facility.
3. **Cost benefit analysis – one omics done well can be much better than many done badly:** Whilst the availability of multiple omics platforms makes multi-omics an appealing option, resources are still limited and must be utilised most effectively. Omics studies are typified by a high number of molecular features for a very small number of samples (p >> n), and this creates challenges when machine learning techniques are applied. Furthermore, omics data are often noisy and burdened by batch effects, both of which become easier to deal with as the sample size is increased and replicate measurements are added. Thus, it is not always a bad idea to sacrifice the number of omics layers or molecular features measured for the sake of sample size and many replicates. Finally, please think about the precision of your method, this is crucial.
4. **Make use of publicly available datasets, and by doing so, experience the beauty of reporting and data standards.** Following the explosive development of omics platforms there is now a rapidly increasing number of biological data available, much of which has not been fully analysed (or analysed at all). This is unlikely to change in the immediate future, and so we discourage conducting a multi-omics experiment in the hope that someone else will perform comprehensive computational analyses at a later point (which might never happen). Conversely, there are also challenges in using available datasets, particularly when integrating different datasets, as this requires highly specialised models in order to deal with batch effects and differences in experimental protocols. Another good reason for re-using others datasets before creating your own is that it is quite instructive of how important it is to adhere to community standards concerning data types and reporting guidelines.
5. **Good publication practice: Resist the temptation to overuse the power of the example, even if (at present) you (might still) convince Reviewers and Editors.** The majority of multi-omic papers start with the generation of a multi-layer dataset, but then go on to pick out just a single example and to finish the study as it it were a very classic molecular biology paper which was explicitly targeted towards this example from the beginning. In the end, such a strategy renders the beauty and the power of multi-layer biology obsolete. However, it is clear as multi-omic studies become more widespread that such ‘nice stories’ are often turning out to be nothing more than to be statistically insignificant correlations between two parallelly occurring phenomena in huge datasets. With more statistical knowledge available across the biological disciplines, the scientific alertness to distinguish causality from correlation is increasing rapidly, and the tide is turning. Multiple fields in other branches of science have already benefited from the application of data science techniques, such as machine learning, to generate predictive models of complex natural phenomena, as will it be in biosciences in the near future. In other words, multifactorial relationships are becoming increasingly accepted as scientific results at the expense of a lower acceptance for scientific oversimplifications.

Alt-text: Box 1
